# Feasibility, reliability and validity of a questionnaire on healthcare consumption and productivity loss in patients with a psychiatric disorder (TiC-P)

**DOI:** 10.1186/1472-6963-13-217

**Published:** 2013-06-15

**Authors:** Clazien Bouwmans, Kim De Jong, Reinier Timman, Moniek Zijlstra-Vlasveld, Christina Van der Feltz-Cornelis, Siok Swan Tan, Leona Hakkaart-van Roijen

**Affiliations:** 1 Department of Medical Psychology & Psychotherapy, Erasmus University Medical Center Rotterdam, Rotterdam, The Netherlands; 2 Netherlands Institute of Mental Health and Addiction (Trimbos-institute), Utrecht, The Netherlands; 3 Academic Centre ‘Geestdrift’Tranzo,Tias Building Room T620, Tilburg University, PO Box 90153, 5000 LE Tilburg, The Netherlands; 4 Institute for Medical Technology Assessment, Erasmus University Rotterdam, PO Box 1738, 3000 DR Rotterdam, The Netherlands; 5 Clinical Center for Body, Mind and Health, GGzBreburg, Tilburg, The Netherlands

**Keywords:** Patient-reported questionnaire, Healthcare utilization, Productivity losses, Reliability, Validity

## Abstract

**Background:**

Patient self-report allows collecting comprehensive data for the purpose of performing economic evaluations. The aim of the current study was to assess the feasibility, reliability and a part of the construct validity of a commonly applied questionnaire on healthcare utilization and productivity losses in patients with a psychiatric disorder (TiC-P).

**Methods:**

Data were derived alongside two clinical trials performed in the Netherlands in patients with mental health problems. The response rate, average time of filling out the questionnaire and proportions of missing values were used as indicators of feasibility of the questionnaire. Test-retest analyses were performed including Cohen’s kappa and intra class correlation coefficients to assess reliability of the data. The construct validity was assessed by comparing patient reported data on contacts with psychotherapists and reported data on long-term absence from work with data derived from registries.

**Results:**

The response rate was 72%. The mean time needed for filling out the first TiC-P was 9.4 minutes. The time needed for filling out the questionnaire was 2.3 minutes less for follow up measurements. Proportions of missing values were limited (< 2.4%) except for medication for which in 10% of the cases costs could not be calculated. Cohen’s kappa was satisfactory to almost perfect for most items related to healthcare consumption and satisfactory for items on absence from work and presenteeism. Comparable results were shown by the ICCs on variables measuring volumes of medical consumption and productivity losses indicating good reliability of the questionnaire.

Absolute agreement between patient-reported data and data derived from medical registrations of the psychotherapists was satisfactory. Accepting a margin of +/− seven days, the agreement on reported and registered data on long-term absence from work was satisfactory. The validity of self-reported data using the TiC-P is promising.

**Conclusions:**

The results indicate that the TiC-P is a feasible and reliable instrument for collecting data on medical consumption and productivity losses in patients with mild to moderate mental health problems. Additionally, the construct validity of questions related to contacts with psychotherapist and long-term absence from work was satisfactory.

## Background

Economic evaluations examine both costs and benefits of alternative healthcare interventions and are increasingly used to inform decision-makers for reimbursement in healthcare. Due to the international interest in economic evaluations, transparency and generalizability of the results are important issues. Additionally, the validity of evaluations also depends on the method of collecting data.

Several arguments support the adoption of a societal perspective in performing economic evaluations [1]. A number of national health economic guidelines recommend performing these evaluations from a societal perspective, including all costs, regardless of who bears the costs and who receives the benefits [2,3]. So, besides the direct medical costs also direct and indirect non-medical costs should be included. Direct costs outside the healthcare sector are directly related to the intervention, but generally do not incur within the formal healthcare system, such as travelling costs and time costs of patients and their informal caregivers. Indirect costs outside the healthcare sector are costs incurred outside the scope of the formal healthcare system arising as a secondary effect of the intervention, such as productivity costs due to absence from work or reduced efficiency during paid or unpaid work [4,5].

Quantities of resources are often collected alongside clinical studies by means of the traditional ‘case report form’ (CRF). The advantage of clinical studies is that the quantities of resources and effectiveness data can be collected simultaneously. However, the data collection is restricted to direct medical costs of the intervention. Thus, other medical cost that may be related to patients’ health and direct and indirect non-medical costs need to be collected from alternative data sources. Administrative databases of healthcare institutions, such as hospitals, other healthcare providers, occupational health institutions, and insurers, are considered to contain the most accurate patient-level data. However, an important limitation of these databases is that they commonly serve other purposes than economic evaluations and the accessibility of these databases may be limited [6]. Furthermore, data needs to be collected from multiple sources which necessitate the cooperation of different institutions and patients’ informed consent at each of these institutions [7,8]. In addition, registrations may be incomplete. For example, databases of health insurers are restricted to the healthcare interventions that are covered by the health insurance system and registrations of occupational health institutions only comprise data on sick leave. More importantly, routine data supporting the cost calculation of informal care and reduced efficiency during paid work are commonly not recorded.

In economic evaluations, data on resource use may also be collected directly from the patient by means of patient self-reports (e.g. diaries, interviews or questionnaires). The reliability of the collected data, however, may be compromised by the degree to which patients recall quantities of resources consumed [9,10]. Furthermore, the validity of the collected data may be restricted by lack of standardized instruments which jeopardizes the meaningful comparison of economic evaluations [7].

The questionnaire on healthcare consumption and productivity losses for patients with a Psychiatric disorder (TiC-P) is a comprehensive questionnaire focused on establishing direct medical costs and productivity costs due to absence from work or reduced efficiency during paid or unpaid work and is widely used in the Netherlands for economic evaluations in mental health [11-14]. In this paper, the performance of the TiC-P is evaluated by addressing the following issues: i) testing the feasibility (practicality) of the questionnaire; ii) assessing the reliability of the questionnaire; and iii) assessing the construct validity of the TiC-P.

## Methods

### TiC-P

The TiC-P is questionnaire designed for self-report in patients with a mental disorder. A translation in English of the questionnaire is available at http://www.imta.nl[15]. The TiC-P is a generic questionnaire, meaning that the items are not related to a target disease. Distinguishing between healthcare consumption and production losses as a consequence of the target disease and comorbidity is difficult, especially in psychiatric disorders, as patients also may have physical symptoms that are connected to the psychiatric illness. Moreover, psychiatric comorbidity is a common occurrence in psychiatric illness.

Before the introduction of the TiC-P the questionnaires’ feasibility in daily practice was assessed by interviewing 20 respondents with a psychiatric disorder who were treated in a specialisedcentre for psychiatry. This resulted in a number of textual changes [16].

The TiC-P consists of two parts, both can also be used separately. Additionally, a number of general questions may be added for collecting data on respondents’ demographic characteristics and co-morbidity.

**Figure 1:**
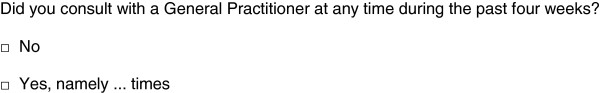
Example of an item on medical resource use.

The first part of the TiC-P includes 14 structured no/yes questions on relevant medical resource items each followed by a question on the volume of medical consumption (see Figure 1).

The questions include contacts within the mental healthcare sector (regional mental healthcare organisation, psychiatrist/psychologist or psychotherapist in private practices or outpatient hospital, institutional day-care treatment, Consultation Agency for Alcohol and Drug addiction (CAD), self-help group and contacts with general healthcare providers (general practitioner, paramedical and social worker, alternative practices, outpatients visits to medical specialists, hospital admission and contacts with an occupational practitioner) and the use of medication. The inclusion of questions related to different types of contacts in mental healthcare makes the TiC-P suitable for broad application, i.e. for various psychiatric disorders. Additionally, depending on the relevance for the target population the questionnaire allows adding or leaving out specific items of resource utilisation.

**Figure 2:**
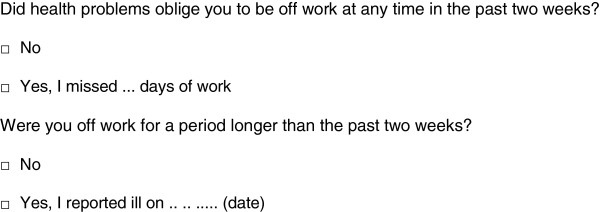
Questions related to absence from work.

Part two consists of the Short Form-Health and Labour Questionnaire (SF-HLQ), a generic instrument to collect data on productivity losses due to health problems and is based on the HLQ [17]. The SF-HLQ aims to measure absence from work and reduced efficiency of paid and unpaid work. Absence from work is measured by two questions related to short-term absence and long-term absence (< 2 weeks and > 2 weeks respectively ) from work, see Figure 2.

**Figure 3:**
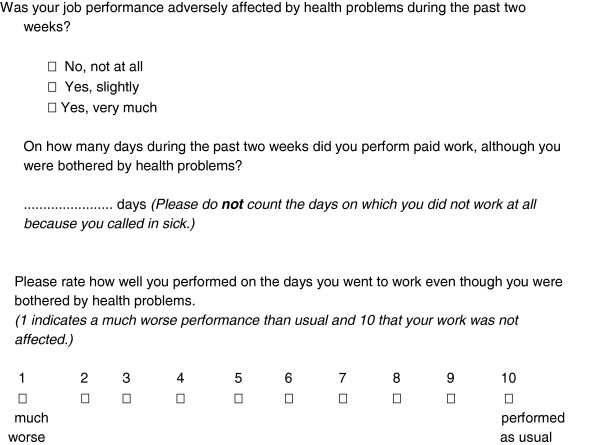
Questions related to reduced efficiency during work.

Additionally, three questions are included for measuring productivity losses due to reduced efficiency during paid work (see Figure 3).

### Study design and subjects

The performance of the TiC-P was assessed using data from two unrelated patient groups with mental disorders in two different studies. Firstly, the study of the TiC-P’s performance was accommodated as part of a Dutch cost-utility study performed alongside a randomized clinical trial evaluating alternative feedback mechanisms during psychotherapy in patients treated in ambulant mental healthcare treatments settings private practice or a regional mental healthcare institution. We refer to this study as the Monitoring Study. The study was approved by the Medical Ethical Committee of the Erasmus University Medical Centre (NL11529.078.06). The study started in 2006 and included a total of 891 patients starting psychotherapy. Inclusion of new patients ended in January 2011 [18]. The TiC-P was used to collect data on medical resource use and productivity losses. The TiC-P was filled out at baseline and monthly during the first 3 months of the study, and every 3 months thereafter. The follow-up ended at treatment completion. In this study a recall period of 4 weeks was applied for collecting data on medical consumption and 2 weeks for collecting data on productivity losses. Generally, the questionnaire was filled out online by the patient at the healthcare setting where psychotherapy was provided.

For practical reasons, data from a second study were obtained to assess the construct validity of long term absence from work data of the TiC-P. This study is referred to as the Collaborative Care Study. The study was approved by the Medical Ethical Committee and the EMGO scientific committee of the VU University Medical Centre (ISRCTN 78462860). In this study patients with a major depressive disorder were randomized to usual care or collaborative care in an occupational healthcare setting [19]. Patients were recruited from workers who were on a sick list for between 4 and 12 weeks. Patient’ characteristics of both studies are presented in Table 1.

**Table 1:** Characteristics of the study populations

	**Monitoring study (n=631)**	**Collaborative care study (n=126)**
Age (mean)	37.7	42.6
Female (%)	66.8	54.0.
Education (%)		
Low	6.9	31.4
Secondary	43.3	33.1
High	49.9	35.5
Diagnoses (%)		
Mood disorder	27	100*
Adjustment disorder	21	
Anxiety disorder	14	
Relational problems	14	
Other^1^	24	

* major depressive disorder; ^1^ Other disorders included: disorders usually first diagnosed in infancy, childhood or adolescence, impulse control disorders, eating disorders, dissociative disorders, sexual disorders, substance-related disorders and psychotic disorders (in order of frequency).

### Analyses

Feasibility and reliability were evaluated using data from the Monitoring study. Feasibility included the TiC-P response rate, respondent’s time of filling out the TiC-P and data completeness on the items of the questionnaire. Completeness is reported as proportions of missing values. Additionally, completeness on reported medication was evaluated including the medication name, the dose per intake, the number of doses per day, and the number of days that the medication was taken during the previous 4 weeks. For this evaluation a random sample (n=283) was taken from the questionnaires submitted by patients who actually reported use of medication. Completeness on medication name was checked manually and was defined missing if no name was reported or in cases that the medication was described in general terms (f.e. sleeping pill, antibiotic). Respondents’ time for filling out the TiC-P was assessed as follows. Patients in the Monitoring study filled out online a number of different questionnaires at the same time. Consequently, information was available of the time of filling out for successive submitted questionnaires. The average time for filling out the TiC-P was calculated by subtracting the times of these questionnaires according to the web-based dataset. Identically, we estimated the mean time for filling out the TiC-P of the first measurement and for the next measurements. Differences in time for filling out the TiC-P at baseline and during follow-up measurements were evaluated using a paired sample *t-*test.

Reliability was assessed using a test-retest design. Test-retest reliability analyses were performed to evaluate consistency of the data reported. For these analyses a subsample was invited to fill out the TiC-P again (retest) two weeks after submission of the original measurement. A cover letter explained the purpose of the retest and we offered a gift voucher of €10 if the retest questionnaire would be returned. Consistency of categorical (yes/no) variables was assessed with percentages of absolute agreements indicating the proportion of cases with the same value on the test and retest questionnaire. To adjust for the fact that a number of these agreements may arise by chance alone, chance-corrected agreements were assessed using Cohen’s kappa coefficients (κ values). The following values were attached to the coefficients: modest (0.21-0.40); moderate (0.41-0.60); satisfactory (0.61-0.80) and almost perfect (0.81-1.00) [20]. Consistency of data on interval level was evaluated by computing intra class correlation coefficients (ICC) (two-way mixed models; absolute agreement).

The construct validity of the TiC-P was evaluated by assessing the agreement with reported and registered data for the items ‘contacts with a psychotherapist’ and ‘long-term absence from work’ including the percentage absolute agreement, absolute differences between reported data en registered data and Spearman rank correlation coefficient (rho). Reported data on contacts with therapists were compared with registration data of the therapists. Additionally, reported data on long-term absence from work was compared to registration data from the occupational health service. As registration data on absence from work of the Monitoring study were not accessible, the latter were derived from the Collaborative Care Study [19]. All statistical analyses were performed in SPSS (V. 17.0; Chicago, IL). Significance was set at a *p*-value of 0.050.

## Results

### Feasibility

The gross response rate of the TiC-P was 72% meaning nearly three quarters of the respondents submitted at least one TiC-P questionnaire during the study.

Overall, the time for filling out was on average 7.8 minutes (SD 5.1). The average time measured for filling out the first TiC-P was 9.4 minutes (SD 5.5) and significantly less for questionnaires that were filled out during follow-up measurements (7.1 minutes; SD 4.7; p < 0.001). Overall, proportions of missing values related to the quantification of medical resource use and productivity losses were small. The most frequently missing information concerned the number of hospital outpatient contacts (2.3%).

To calculate the costs of medications, 4 items are required: i.e. the name of the medication, dose per intake, the daily dose and the number of days that the medication was used. In 29% of cases, at least one of these items was missing. Table 2 presents the share of missing values per item. Most frequently missing information concerned the name of the medication and the dose per intake. For specified medications, substituting missing data concerning the dose of intake with daily defined doses (DDD) decreased the number of cases of missing cost data for this item to 10.2%.

**Table 2:** Completeness of information on medication (n=283)

	**Missing (%)**
Specification of medication	12.4
Dose per intake	20.2
Number of intake per day	2.8
Number of days of medication intake	
during the preceding 4 weeks	4.6

### Reliability

A total of 111 respondents filled out the retest questionnaire (response rate retest 61.8%). Of these, 99 retest questionnaires were eligible to assess consistency of the data reported. The period between test and retest questionnaire of the remainder 12 respondents was more than one month and was considered too large for assessing the reliability. The retest was submitted on average 17 days (SD 7.7; range 1–28) after submission of the initial questionnaire.

Absolute agreement on medical resource use (yes/no questions) ranged from 82% to 99%. Agreement according to Cohen’s kappa of items related to medical consumption was as follows: almost perfect n=2; satisfactory n=6; moderate n=4. Generally, ICCs of test-retest measurements on the number of contacts with the healthcare providers showed good agreement. Non-significant agreement was found on measurements related to the number of contacts with a social worker, medical specialist and alternative medicine practice. Due to the small number of patients reporting on the number of contacts with specific healthcare provider ICCs of 5 items (e.g. the duration of hospital admission, number of hospital day-care treatment, contacts with a self help group and CAD, and outpatient visits to mental healthcare providers) could not be calculated (see Table 3).

**Table 3:** Reliability: consistency of data on medical resource use (n=99)

	**Absolute agreement (%)**	**Cohen’s Kappa**	**Use of service (%)**	**ICC (95% CI)**
General practitioner	81.8	0.597	25.3	0.804
				(0.608-0.908)
Therapist in regional mental healthcare	91.9	0.649	12.1	0.769
				(0.341-0.929)
Mental healthcare therapist with private practice	88.9	0.618	75.8	0.740
				(0.613-0.829)
Mental healthcare therapist hospital outpatient	0.0	-	1.0	*
Occupational physician	88.9	0.600	12.1	0.884
				(0.652-0.965)
Hospital outpatient visit to a medical specialist	96.0	0.645	4.0	0.000
				(−0.878-0.878)
Paramedical healthcare provider	93.9	0.805	16.2	0.825
				(0.569-0.935)
Social worker	97.0	0.711	4.0	0.571
				(−0.475-0.965)
Alternative medicine practice	94.9	0.519	6.1	0.231
				(−0.164-0.775)
Self help group	98.0	0.492	1.0	*
Consultation Agency Alcohol & Drugs (CAD)	100.0	-	1.0	*
Hospital day care treatment	99.0	0.795	2.0	*
Hospital admission duration?	99.0	0.662	2.0	*
Use of medication	91.9	0.839	49.5	0.684
				(0.499-0.810)

* Too few cases to analyze; - Not applicable.

Agreement between test and retest on nominal variables related to productivity losses was satisfactory. Almost perfect consistency was found on the reported number of sick leave days during the preceding 2 weeks (ICC 0.83; CI 0.55-0.94). Consistency on the number of days at work while impeded between test and retest measurement was moderate. However, consistency of the efficiency rates at work was satisfactory (see Table 4).

**Table 4:** Reliability: consistency of data on productivity losses (paid work: n=79)

	**Absolute agreement (%)**	**Cohen’s Kappa**	**Yes (%)**	**ICC (95% CI)**
Were you on sick leave?	87.3	0.654	25.3	-
Number of days off work during last 2 weeks			25.3	0.825
				(0.553-0.940)
Sick leave episode > 2 weeks?	93.7	0.762	11.1	-
Were you impeded during paid work?	81.3	0.646	-	-
Number of days at work while impeded	-	-	27.3	0.556
				(0.224-0.771)
Efficiency rate* (at work while impeded)	-	-	27.3	0.729
				(0.492-0.866)

- Not applicable; * Rate ranging from 1 to 10 indicating much to no influence of health problems on work performance.

### Construct validity

The construct validity of part one of the TiC-P was evaluated by comparing the patient reported number of contacts with psychotherapist of 114 responders (corresponding to a total of 365 measurements) with the registration of the psychotherapists. The reported number of contacts in the preceding 4 weeks was 1.9 (SD 1.5) ranging from 0 to 8 and was highly correlated with the number of contacts according to the registration data (rho= 0.791). Absolute agreement between the number of reported and registered contacts was 76.7%. The difference between reported and registered data was on average 0.01 (SD 0.97). Occupational registry data and reported data of absence from work data were available of 117 respondents derived from the Collaborative Care Study. Absence from work was on average 274 (calendar) days (SD 167). Absolute agreement on reported and registered data was 54.2%. Accepting a margin of +/− 7 days as acceptable between the reported and registered date of onset of absence from work resulted in an agreement of 70.9%. The difference between reported and registered date of absence from work was on average 5.7 days (SD 20.6). The correlation between reported and registered days of absence was 0.663.

## Discussion

In this study the feasibility and reliability of the TiC-P were evaluated. Additionally, the construct validity of two items of the TiC-P was assessed. The small number of missing data indicated that the questionnaire was generally well understood. The response rate was at an acceptable level (72%). The average completion time of the questionnaire (on average below 10 minutes) was acceptable. The test-retest analyses showed good agreement on items of medical resource use that were frequently reported by the respondents. Absolute agreement between reported data on contacts with psychotherapists and long-term absence from work and registration data was around 70-75%. Additionally, the construct validity of the items on contacts with psychotherapists and long-term absence from work was satisfactory.

Thus, the TiC-P, including the SF-HLQ, seems a feasible and reliable instrument for measuring healthcare utilization and productivity loss. Additionally, the findings regarding the construct validity of the items ‘contacts with psychotherapist’ and ‘long term absence from work’ are promising.

Qualitative feedback on the questions was evaluated on the draft version of the TiC-P. For the current study, feasibility was operationalized as the response rate, the time for filling out the questionnaire and data completeness. Generally, patients in the Monitoring study filled out the questionnaire at the healthcare setting. This may have positively influenced the response rate and data completeness of the questionnaire in comparison to filling out the TiC-P at home. Proportions of missing values on the items were small with exception of missing values related to the measurement of the use of medication. The findings on incompleteness of self-reported data on medication are in line with findings in another study among elderly patients [21]. Cost calculation of medication requires relatively comprehensive information of the respondents. It may be feasible to reduce the number of questions on medication using daily defined dose for the calculation of the costs. If costs of medication are expected to contribute substantially we recommend considering alternative sources for collecting these data, e.g. patient records.

The relatively easy way of filling out questionnaires online may present an underestimation of the time needed for filling out the paper version of the questionnaire. Also, the relatively young population participating in the Monitoring study may underestimate the time to fill out the TiC-P in general. Additionally, in the online version respondents were pointed out to the missing quantification in case of reporting ‘yes’ on the items. Despite this, respondents were able to ignore the automatic signal and continue the questionnaire. However, this might have decreased the number of missing values.

Consistency between test and retest measurement was relatively high. Generally, the reliability of test retest analyses require similar circumstances of the successive measurements and an appropriate time interval between the measurements moments. For this study we chose to send the retest questionnaire after t=1 (the second measurement) since we assumed that changes in medical consumption and productivity losses are limited at the start of therapy. A number of 180 respondents were invited for filling out a retest questionnaire. The response rate was relatively low. Additionally, 10% of the retest questionnaires were filled out after a relatively long period. Due to the relatively small sample of the retest, the generalizability and the interpretation of the results warrant some caution. Despite of this, the ICCs related to contacts with healthcare providers that were contacted most frequently (i.e. > 12% of the respondents) seem satisfactory and have relatively small confidence intervals. A number of healthcare providers who were contacted less frequently (e.g. contacts reported by < 12% of the respondents) had lower agreement scores. Consequently, whether the frequencies of these contacts are relatively constant over time may be argued. More research is necessary to further assess the reliability of these items.

The reliability of short-term absence from work was satisfactory. Test and retest figures related to the number of days at work while impeded by health problems were moderate. It can be assumed that it is more complicated to remember the exact number of days at work with impediment. However, alternative methods for measuring reduced efficiency are currently not available. More research on this topic is warranted.

Agreement between reported and registered data on the number of contacts with psychotherapists was satisfactory, indicating an acceptable construct validity of this item. This finding is in line with other studies indicating that self-reports provide accurate data for more important and for less frequent events [9,22]. It was not possible to study resource utilization other than contacts with psychotherapists who participated in the clinical study. Consequently, further research is necessary for assessing the construct validity of measuring the other items of health care utilization. Agreement between the number of days of absence from work based on data derived from the occupational health service and self-report of the patient was satisfactory. Our results are in line with previous studies of patient-reported absence from work [23-25]. A limitation of our study is that we were only able to compare reported data with registered data on long-term absence from work. However, Severens et al. [23] found that 95% of patient reported data matched the registered data on absence from work perfectly applying a recall period of 2 and 4 weeks.

Another limitation is that our study was performed in patients with mental disorders treated in ambulant settings e.g. among patients with less severe mental disorders. We expect that the findings in our study can be generalized to other groups of patients. However, future research on this is desired. Currently, the default version of the TiC-P uses a recall period of three months for measuring medical consumption and one month for measuring productivity losses is applied. This interval is in line with commonly applied measurement intervals in clinical studies. In the Monitoring study, a recall period of four and two weeks was applied respectively. This may limit the generalizability of the results of this study for the current version of the TiC-P. Further research should indicate the impact of a longer recall period on the different items of the TiC-P.

Finally, the current version of the TiC-P is translated by a professional language institution into a patient-friendly version using more simple language. We assume that this will enhance the feasibility and validity of the TiC-P.

## Conclusions

In economic evaluations, data on resource use is frequently collected directly from the patient by means of patient self-reports. Therefore, the reliability and validity of the thus collected data are important for the validity of the evaluation. Additionally, the application of standardized instruments allows a meaningful comparison of economic evaluations.

The results of our study indicate that the TiC-P is a fairly feasible and reliable alternative for collecting data on resource use data in comparison to collecting data from registries. Additionally, the results on the validity of the reported data related to contacts with psychotherapists and long term absence from work for the validity of the TiC-P are promising.

## Competing interests

The authors declare that they have no competing interests.

## Authors’ contributions

RT and LH were project leaders of the Monitoring Study and contributed to the study design and monitoring of the study. CFC contributed to the study design and monitoring of the Collaborative Care Study. KdJ, MV and SST were involved in the collection of data. CB wrote the manuscript and performed the analyses. All authors contributed to the interpretation of the analyses and commented on the manuscript. All authors read and approved the final manuscript.

## Pre-publication history

The pre-publication history for this paper can be accessed here:

http://www.biomedcentral.com/1472-6963/13/217/prepub
